# Publisher Correction: A computational reward learning account of social media engagement

**DOI:** 10.1038/s41467-021-22067-6

**Published:** 2021-03-16

**Authors:** Björn Lindström, Martin Bellander, David T. Schultner, Allen Chang, Philippe N. Tobler, David M. Amodio

**Affiliations:** 1 Department of Psychology, University of Amsterdam, Amsterdam, The Netherlands; 2 Center for Psychiatry Research, Department of Clinical Neuroscience, Karolinska Institutet, Stockholm, Sweden; 3 Department of Psychological and Brain Sciences, Boston University, Boston, MA USA; 4 Zurich Center for Neuroeconomics, Department of Economics, University of Zürich, Zürich, Switzerland; 5 Department of Psychology, New York University, New York, NY USA

**Keywords:** Human behaviour, Communication, Human behaviour

Correction to: *Nature Communications* 10.1038/s41467-020-19607-x, published online 26 February 2021.

The original version of this Article was updated shortly after publication, because the Reporting summary file was incorrect. The error has now been fixed and the Reporting summary PDF is available to download from the HTML version of the Article.

The original version of this Article contained errors in the Data availability statement, which read:

“The data for Study 1 are available from ref. 30. Data for Study 2 and Study 3 are available at the Open Science Framework (osf.io/765py/). A reporting summary for this article is available as a Supplementary Information file. Source data are provided with this paper”.

The corrected version states:

The availability of the dataset analyzed in Study 1 is described in Ferrara, E., Interdonato, R. & Tagarelli, A. Online popularity and topical interests through the lens of instagram. In *Proc. 25th ACM Conference on Hypertext and Social Media—HT’14*, 24–34 (ACM Press, 2014).

The datasets analyzed in Study 2 are available on reasonable request from the corresponding author. The dataset analyzed in Study 3 is available on the Open Science Framework (https://osf.io/765py/). A Reporting summary for this article is available as a Supplementary information file. Source data are provided with this paper”.

This has been corrected in both the PDF and HTML versions of the Article.

