# Supplementary material for: A computational reward learning account of social media engagement
Source: Nat Commun. 2021 Feb 26;12:1311. doi: 10.1038/s41467-020-19607-x (PMC7910435; doi:10.1038/s41467-020-19607-x)
Supplement: Supplementary file 1 — Supplementary Information [file 41467_2020_19607_MOESM1_ESM.pdf]

Supplementary Information for

A computational reward learning account of social media engagement

Björn Lindström[1], Martin Bellander[2], David T. Schultner[1], Allen Chang[3], Philippe N. Tobler[4], David M. Amodio[1,5]

1. Department of Psychology, University of Amsterdam, Amsterdam, The Netherlands
2. Center for Psychiatry Research, Department of Clinical Neuroscience, Karolinska Institutet, Stockholm, Sweden
3. Department of Psychological and Brain Sciences, Boston University, Boston, Massachusetts, USA
4. Zurich Center for Neuroeconomics, Department of Economics, University of Zürich, Switzerland
5. Department of Psychology and Neural Science, New York University, New York, NY, USA

Corresponding author:

Björn Lindström
University of Amsterdam
Department of Psychology
Postbus 15900
1001 NK Amsterdam
The Netherlands
bjorn.r.lindstrom@gmail.com

Table of contents

Supplementary Methods 4

Supplementary Table 1. Descriptive statistics for likes, τ_{Post}, and followers across the four datasets of Study 1 & 2........ 4

Additional information about Study 1 4

Additional information about Study 2 5

Computational modeling 5

Simulation of key empirical regularities in operant conditioning research........ 5

Supplementary Figure 1. The RL model reproduces key empirical regularities in operant conditioning research 7

Model estimation......... 8

Time distribution of likes........ 8

Model recovery........ 9

Supplementary Figure 2. Model recovery. 9

Generative Model Simulations........ 9

Supplementary Figure 3. AICW distributions........ 10

Supplementary Figure 4. Additional example individuals in Study 1 (Instagram)......... 11

Experimental manipulation of social reward rates........ 11

Methods 11

Supplementary Figure 5. Overview of the experimental design. 13

Statistical analysis and data exclusions........ 13

Supplementary Notes........ 14

Supplementary Note 1 14

Supplementary Note 2 15

Supplementary Note 3 16

Supplementary Note 4 16

Supplementary Table 2. Comparison of utility functions in Study 2 showed, in contrast to Study 1, no evidence for diminishing marginal utility of likes in reinforcement learning........ 18

Supplementary Note 5 18

Supplementary Note 6 19

Effect of time dependent terms........ 19

Supplementary Table 3. Comparison of the RL model to alternative learning models without time-dependent effort- and opportunity cost terms 19

Alternative effort cost formulations........ 20

Supplementary Table 4. Comparison of the RL model to alternative effort cost formulations 20

Effect of instrumental policy 21
Supplementary Table 5. Comparison of the RL model to alternative effort cost formulations 21
Comparison with a model based on foraging theory 21
Supplementary Table 6. Comparison of the RL model to the F-model 23
Supplementary Note 7 23
Supplementary Note 8 23
Supplementary Table 7. Regression estimates for the effect of R in different regression models.. 25
Supplementary Note 9 25
Alternative social comparison models 26
Supplementary Table 8. Comparison of the ξ + RL model to alternative social comparison definitions 27
Supplementary Note 10 27
Robustness analysis 27
Social comparison does not change cluster structure 27
Supplementary Note 11 28
Robustness analyses 28
Participant-specific reward condition 29
Analysis of individual differences 29
RL model-based analysis 30
Supplementary Note 12 30
Supplementary References 31

Supplementary Methods

	**Likes**				**τ_{Post}**				**Followers**	
**Dataset**	**M Pop.**	**Md Pop.**	**M Ind.**	**Md Ind.**	**M Pop.**	**Md Pop.**	**M Ind.**	**Md Ind.**	**M Pop.**	**Md Pop.**
Study 1: *Instagram*	377	43	338	74	1.82	0.95	3.12	1.45	324	145
Study 2: *Men's Fashion*	17.46	14	16.46	12	14.61	2.99	33.64	6.99	-	-
Study 2: *Women's Fashion*	16.45	16	17.22	16.5	11.96	1.75	24.47	4.55	-	-
Study 2: *Garden*	6.1	4	5.43	4	8.03	1.01	22.2	2.98	-	-

Supplementary Table 1. Descriptive statistics for likes, τ_{Post}, and followers across the four datasets of Study 1 & 2. The table shows both sample averages (Pop.), and individual-level averages (Ind.). The latter was generated by computing the mean (M) and the median (Md) for each individual, and then summarized with mean-of-means and median-of-medians of the individual level averages.

Additional information about Study 1

Inclusion in the original Instagram dataset (collected by[1]) was based on participation in at least one of Instagram's weekly photography contests. Contest participation was denoted by the addition of a hashtag with prefix "*#whp-*" to an Instagram post. All media uploaded by a random selection of 2,100 users with at least one "*#whp-*" hashtag (including media that were not tagged with #whp-hashtags) were gathered and their information retrieved and stored. Study 1 was based on a subset (with at least 10 posts, n = 2,039) of these users.

To quantify contest participation in our dataset, we compared the number of posts with a "*#whp-*" hashtag to the total number of posts, and found that "*#whp-*" hashtags comprised 2.3% of the total number of posts. The number of "*#whp-*" hashtags per user ranged from 1 to 275, with a median of 19 (comprising 0.0005% to 71% of posts). We show below that the number of contest participations did not predict social media behavior (*"No evidence for association between Instagram photo contest participation and social media behavior"*).

Additional information about Study 2

For Study 2, we obtained public data from three topic focused social media forums (Men's fashion: *styleforum.net*, Women's fashion: *forum.purseblog.com*, Gardening: *garden.org*), where users could provide likes to each other as feedback for posts. These forums are organized in "threads" (which users can start) focused on a specific topic or question. Because our focus was how likes affected posting behavior, we focused for simplicity on threads with a high proportion of image posts rather than textual exchange (where many factors other than likes are likely to affect behavior). For this purpose, we selected three high profile threads (with many thousands of posts each), where users primarily posted images of their own clothes, from the Men's fashion forum, and eight threads (primarily on topics related to posting images of users' handbags or shoes) from the Women's fashion forum. On the Gardening forum, we instead opted to acquire all posts from the entire forum, because that forum had provided the possibility for assigning likes to their origin (which allowed tracking the entire learning history of individual users. In the two other forums, we only analyzed posts from the time period where likes were possible). The similarity of results from Instagram (Study 1) and the two fashion forums (where the analyses was based on a subset of threads, Study 2), and from the Gardening forum (where the analyses included all threads, Study 2), indicates that the results are robust to variation in data sampling strategy.

In all three datasets, we removed all posts that did not include user-generated images (in Supplementary Note 5 below, we confirm that results are qualitatively identical when including text-based posts**)** or that quoted other posts, and ordered the posts in sequential order for each individual user. To adjust for potential differences between threads in average posting latency, the statistical analyses included either fixed (Men's and Women's fashion forum) or random (Gardening forum) effects for thread. For simplicity and consistency with Study 1, the model-based analysis did not distinguish between threads. The datasets were anonymized (i.e., no information about the post content or username was retained), and only contained the time stamps and likes associated with each post.

Computational modeling

Simulation of key empirical regularities in operant conditioning research. The $\overline{\text{R}}$L model is based on the normative theoretical framework developed by Niv and colleagues[2] to explain free operant behavior in animals. Because their theory was focused on optimal equilibrium (rather than learning) behavior, designing the $\overline{\text{R}}$L model to fit the dynamics of social media data

required multiple adaptations (e.g., novel updating equations and gradient computations, simplified parametrization, different policy definition). Therefore, we verified that the $\overline{\text{R}}$L model can accurately reproduce the classic, qualitative behavioral patterns of animals trained in Skinner boxes. This is important for establishing the theoretical validity of the $\overline{\text{R}}$L model as a tool for identifying reward learning on social media.

We conducted three sets of simulations, each aimed at reproducing a standard empirical regularity from the operant conditioning literature. Each simulation was ended after the model elicited 200,000 responses, and repeated five times (corresponding to five "artificial rats"). As typical for operant conditioning research[3], we analyze well-learned, "steady state" behavior (the last 100,000 responses). Importantly, the $\overline{\text{R}}$L model was not altered in any way relative to our analysis of social media data.

First, we used our $\overline{\text{R}}$L model to simulate behavior in classic variable interval (where the first response after a pre-specified, random time interval is rewarded) and variable ratio (where each response has a pre-specified probability to be rewarded) schedules of reinforcement[3]. These reinforcement schedules are the cornerstones of free operant behavior. The typical pattern of results is higher responses rates in both (i) interval schedules with shorter, relative to longer, interval durations, and (ii) ratio schedules with lower, relative to higher, ratio requirements. The $\overline{\text{R}}$L model reproduces both patterns of results (Supplementary Figure 1A-B). Furthermore, as expected by theory[2], the model's estimate of the average reward rate, $\overline{\text{R}}$, was consistently positively related to the response rate (i.e., negatively related to the average response latency). Second, we verified that the $\overline{\text{R}}$L model reproduces the key difference between ratio and interval schedules: the response rate on ratio schedules is higher than on interval schedules with matched (yoked) reward rate[3]. The theoretical explanation for this result is that shorter inter-response-intervals (i.e., τ_{Post}) increases the probability of reward in ratio, but not interval, schedules. We first simulated responding on variable ratio schedules, and then used the exact durations between reward deliveries to set the interval durations for the yoked simulation. Again, the $\overline{\text{R}}$L model accurately reproduced this pattern (Supplementary Figure 1C). Finally, we simulated responding on Differential-Reinforcement-of-Low-rates (DRL) schedules[4]. In DRL schedules, the animal has to wait a fixed minimum duration (given by the schedule) since its last response to receive reward. Any premature response resets the schedule, meaning that the animal needs to be able to estimate the interval elapsed since its last response, and time its next response accordingly (as well as suppress the natural tendency to start

responding before the usual time of reward). We find that our $\overline{\text{R}}$L model learns to time the schedule duration (Supplementary Figure 1D). Similar to data from DRL experiments, we find that the standard deviation of the response latencies (i.e., τ_{Post}) is positively related to the length of the target interval (c.f., scalar property of timing[5]). Together, these simulations demonstrate that the $\overline{\text{R}}$L model accurately reproduces key result patterns from the operant conditioning literature, and supports the theoretical validity of the $\overline{\text{R}}$L model as an account of reward learning on social media.

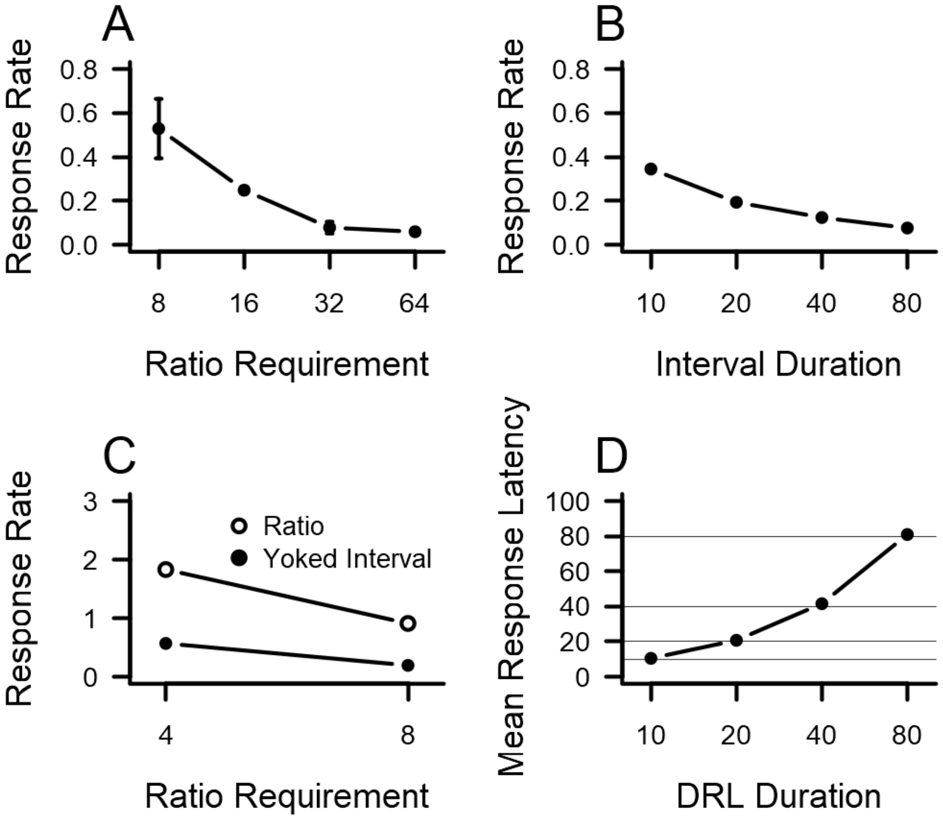

Supplementary Figure 1. The $\overline{\text{R}}$L model reproduces key empirical regularities in operant conditioning research. (A) The simulated response rate (i.e., 1/mean(Response Latency)) is higher on schedules with lower ratio requirements. The ratio requirement refers to the mean number of responses required for receiving reward (each response had P = 1/ratio requirement to be rewarded). Each point is the mean of N = 5 independent simulations, error bars are 1 SE. (**B**) The simulated response rate (i.e., 1/mean(Response Latency)) is higher on schedules with shorter average interval durations (each individual interval was drawn from an exponential distribution with the mean corresponding to the interval duration). The interval is set after each incurred reward. Each point is the average of N = 5 independent simulations (**C**) The response rate (i.e., 1/mean(Response Latency)) is higher on random ratio schedules than interval schedules with matched (yoked) reward rate. Each data point is N = 1 simulation run. (**D**) The model learns the waiting time (DRL duration) required for receiving reward. The mean Response Latency was consistently above the DRL duration. Each point is the average of N =

5 independent simulations. The model parameters were set to $\alpha = 0.001$, $P = 1$, $C = 0.01$ for all simulations.

Model estimation. Computational model estimation was conducted on the individual level using maximum likelihood techniques, with an exponential likelihood function. To avoid local minima in parameter fitting, optimization was initiated with at minimum n_p*10 randomly selected start values, where n_p is the number of free parameters. The Akaike Information Criterion (AIC), which penalizes model complexity, was used for model comparison. The relative model fit was assessed with individual-level AIC weights (AIC_W), which can be interpreted as the probability that a given model, in the candidate set, best accounts for the data[6]. Bayesian model comparison was conducted with the *VBA* package[7], using the AIC as approximation to model evidence.

All variables where initialized at 0, except $\overline{R}$. Within a dataset, the initial value of $\overline{R}$ was set to the median number of likes received for the first post, divided by the median latency between the first and the second post (i.e., $\bar{R}^{t=1}$ = mdn($R^{t=0}$)/mdn($\tau_{Post}{}^{t=1}$) (note that we removed the first post for each user from the analysis of the empirical data, because τ_{Post} is undefined for the first post). Although this formulation uses data from individual *i,* which is also used for model estimation, the contribution from any given individual is negligible. The initial value of $\overline{R}$ was not crucial for the model fit, as preliminary analyses showed that setting it to 0 or the median number of likes at *t=1* produced similar results (although model fit is better if $\overline{R}$ is initialized to a positive value), while estimating the initial value as a free parameter did not reliably improve model fit.

Time distribution of likes. For analytical simplicity, the model analyses assumed that *R* (i.e., the number of likes) received for a post accrued instantaneously. However, in reality the likes provided for a given post are likely to be distributed in time. We assessed the empirical time distribution of likes per post in the Women's fashion dataset (where timestamps for all likes were available) and found that the number of likes followed a heavily skewed and long-tailed distribution. In other words, most likes were provided in close temporal proximity to the post. Testing this quantitatively, we found that the number of likes provided within the first hour of a post was strongly predictive of the total number of likes the post would receive (Spearman's rho = .88, $p < .00001$), indicating that our analytical simplification did not reduce realism.

Model recovery. To assess the ability of our model estimation procedure to differentiate between the $\overline{R}$L model and the No Learning model, our main model-based analysis, we conducted model recovery tests. We simulated each model 1000 times, with randomly drawn parameters (from the unit range), and fitted the simulated data both with the generative model and the alternative model (e.g., simulated the $\overline{R}$L model, and fitted both the $\overline{R}$L model and the No Learning model) using the same methods as for the empirical data. We find that the model comparison procedure recovers the generative model with high likelihood (see Supplementary Figure 2).

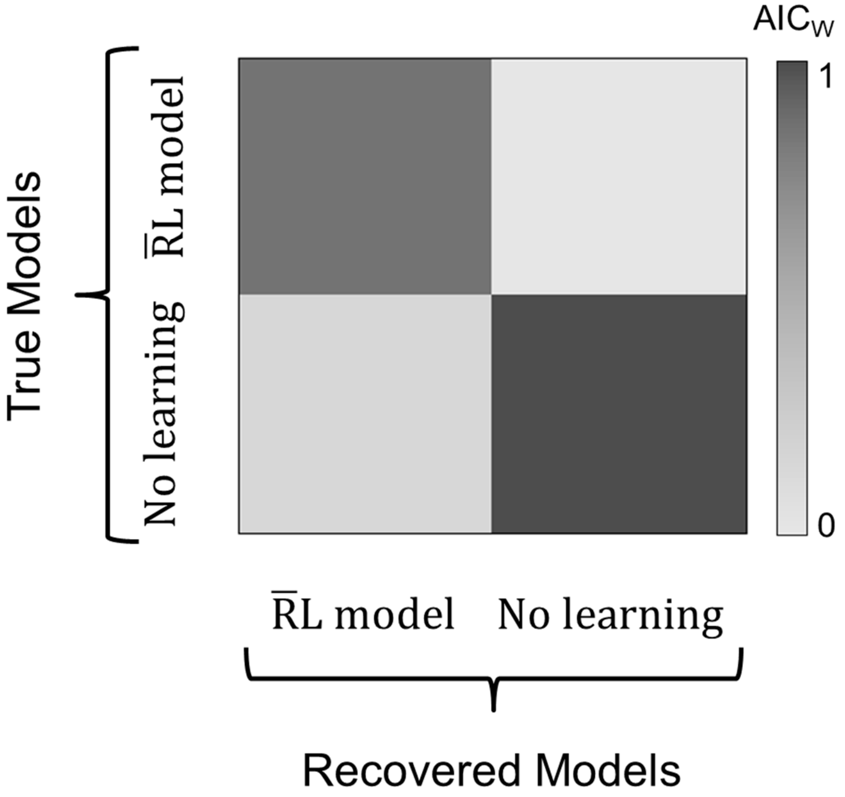

Supplementary Figure 2. Model recovery. The confusion matrix shows the mean AIC_W for the $\overline{R}$L model and No Learning models, conditional on the generative, "true", model (based on 1000 simulation runs of each model).

Generative Model Simulations. Generative model simulations were based on the median estimated parameter values from the respective dataset. For each simulated individual, a simulation run had the same length (i.e., number of posts) as in the real data, resulting in a full simulated dataset of the same dimensions as the empirical dataset. In the main set of simulations, social rewards (i.e., likes) were generated as draws from a Poisson distribution,

with the same mean for each simulated individual. The mean of the Poisson distribution had initial value *i*, and changed across simulation time points with slope *s*. Both *i* and *s* were estimated from the empirical data, for respective data set, using mixed-models with Poisson link function. In these simulations, the expected value of *R* (the social reward) was independent of τ_{Post}. In other words, the reward for any given post was independent of the model policy.

In an alternative set of simulations, we explored the consequence of this assumption by making *R* dependent on $\tau_{Post.}$ In this set up, *R* was maximized for a specific value *M* (e.g., 1 day, which varied randomly across simulations) of τ_{Post}, and decreased exponentially around *M* as a function of the absolute difference between *M* and τ_{Post}. We found that the $\overline{\text{R}}$L model can, with sufficient time, adjust its policy to approximate *M,* and that the expected difference between high and low $\overline{\text{R}}$ is similar to that of the main simulation (results available from corresponding author, c.f., Figure 1E). This shows that the predictions of the $\overline{\text{R}}$L model are not dependent on assumptions of *R*.

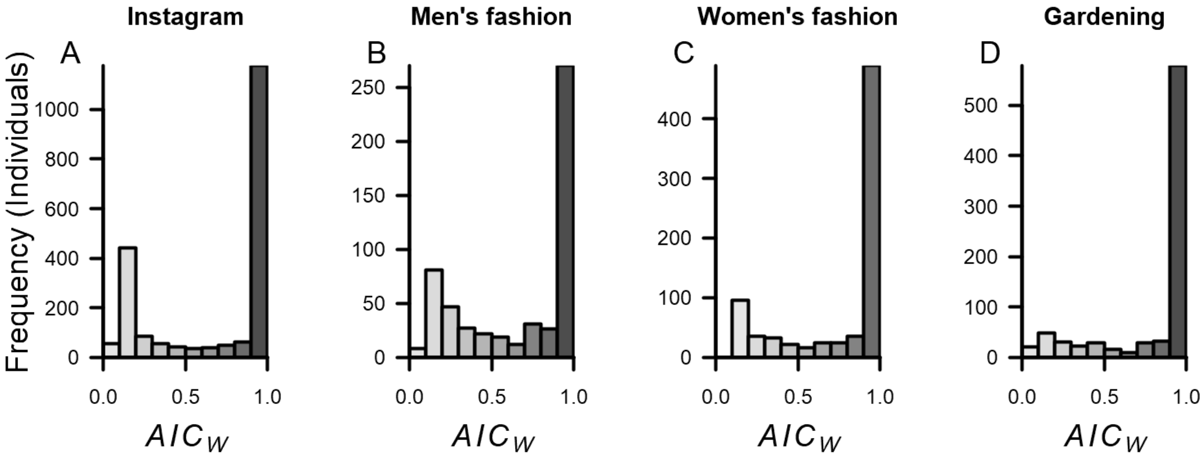

Supplementary Figure 3. AICW distributions. The figure displays the AIC_W in favor of the $\overline{\text{R}}$L model relative to the No Learning model in (A) Study 1 (Instagram, N = 2,039 independent individuals), and in Study 2 (B) N = 543, (C) N = 773, (D) N = 813 independent individuals. Darker color indicates stronger support for the $\overline{\text{R}}$L model. The "spikes" at ~0.2 is explained by the parameter penalty for the $\overline{\text{R}}$L model for individuals where the loglikelihoods of the two models were very similar (within ~1 unit).

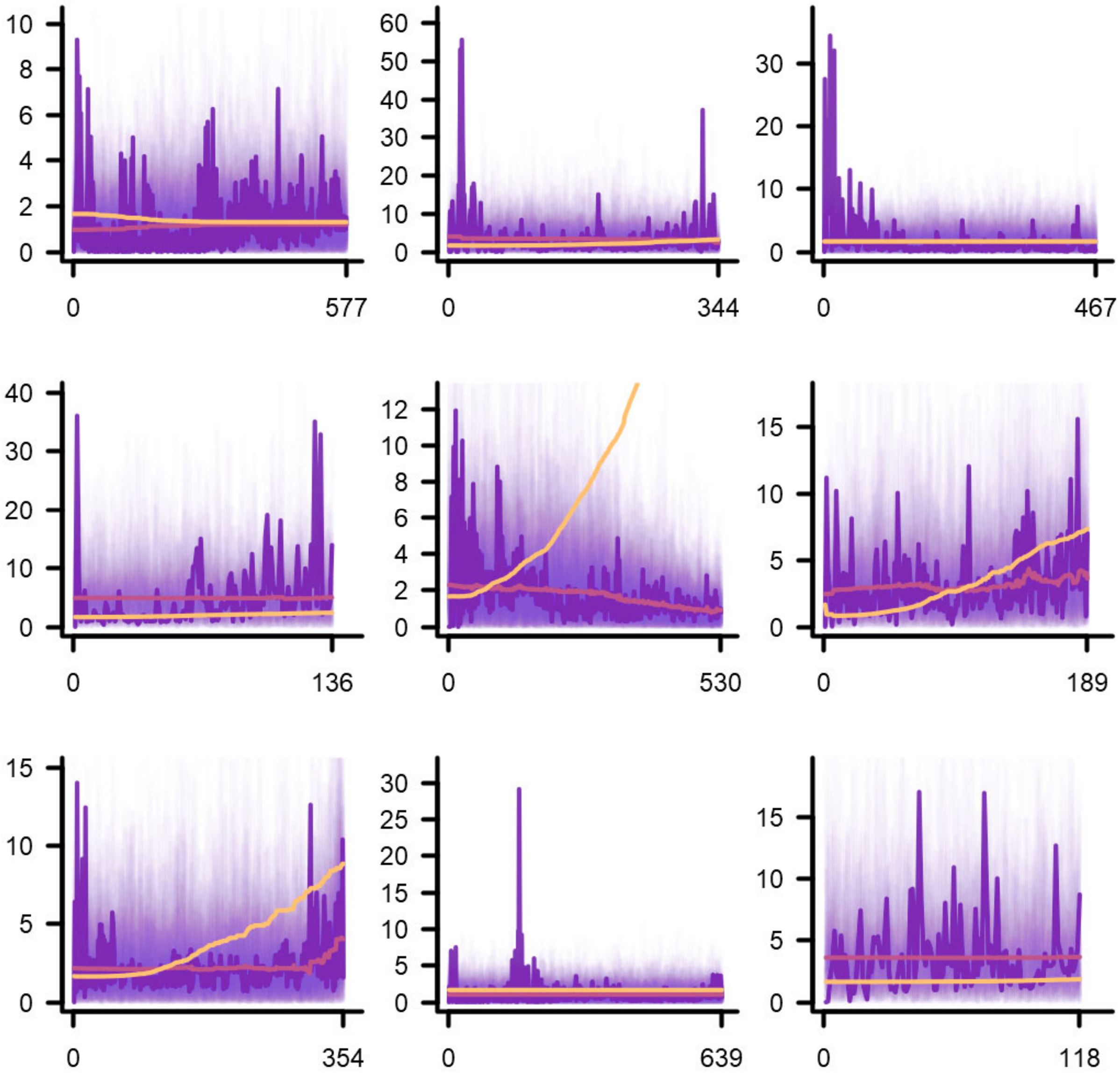

Supplementary Figure 4. Additional example individuals in Study 1 (Instagram). To supplement Figure 2C (main text), we here show nine (out of 2,039) additional randomly selected example individuals. Purple lines indicate τ_{Post}, yellow lines $\overline{R}$, and red lines the model policy/threshold. The faded purple lines show 100 simulations of τ_{Post} from the estimated model policy, which illustrate the expected degree of variability given that policy, and how the empirical τ_{Post} typically falls within this range. The x-axis shows the number of social media posts per individual. These examples show the wide variability in social media posting patterns.

Experimental manipulation of social reward rates

Methods. We invited participants (with minimum 95% approval rate) on Amazon Mechanical Turk to take part in a study on "humor on social media". 179 participants completed the study,

and were payed 3$ in compensation. The study was approved by the ethical review board of the University of Amsterdam, The Netherlands. All participants provided informed consent.

Participants were instructed that they would take part in a study of humor on social media, in which they would interact with 19 other online participants (“users”). To resemble the typical structure of social media, the experiment involved a (simulated) “feed”, where the participant could observe images shared by other users, and “post” (share with the other users) their own images (Supplementary Figure 5). More specifically, the participant could “post” a type of humorous image, known as a “meme” (see Supplementary Figure 5A), that is popular on social media (meme images were downloaded from *https://www.reddit.com/r/memes/* and *https://www.reddit.com/r/dankmemes/*, and screened for objectionable content such as profanity, racism, or sexism). As feedback on the posted images, the participant received social feedback (“likes”) from the other users. The participant could also “like” memes posted by other users (Supplementary Figure 5B), but to preclude social comparison, they could not observe the likes other users received. Unbeknownst to the participant, the responses of the other users were computer-controlled in order to manipulate the likes provided to the participant (participants were fully debriefed upon the publication of this study).

Importantly, to resemble real social media interaction, the experiment had no trial structure. Instead, the participants could post their own memes and provide likes of memes posted by the other users whenever and as often as they wished during the experiment (25 minutes). To post a meme image, the participant selected the image they wanted to share with the other users from a set of six randomly selected images (Supplementary Figure 5A). The purpose of this was to create a sense of self-expression, while also preventing participants from generating unethical or nonsensical content. Next, the participant was asked (but not required) to provide three “informative and descriptive” nouns or adjectives (termed “tags” to correspond to social media terminology) to the image they selected (e.g., “funny”). The purpose of the tags was to associate an effort cost with posting, as typical on real social media platforms. Next, the participant received 0-19 likes (after waiting 14 s, which matched the display duration of the images that were displayed in the “feed”) in response to their meme post. Crucially, the average number of likes was manipulated within participant (low reward: $M = 4.5$ likes per post, uniform distribution 0-9. High reward: $M = 14.5$ likes per post, uniform distribution 10-19; low/high order counterbalanced with random assignment to order condition) in order to test the influence of social reward rate on posting response latencies.

After the experiment, the participants were asked to report how many followers they had on Instagram, Twitter, and Facebook, and how many likes they received on average for a post on the social media platform that they typically used. In addition, we administered the nine-item Social Media Disorder Scale[8].

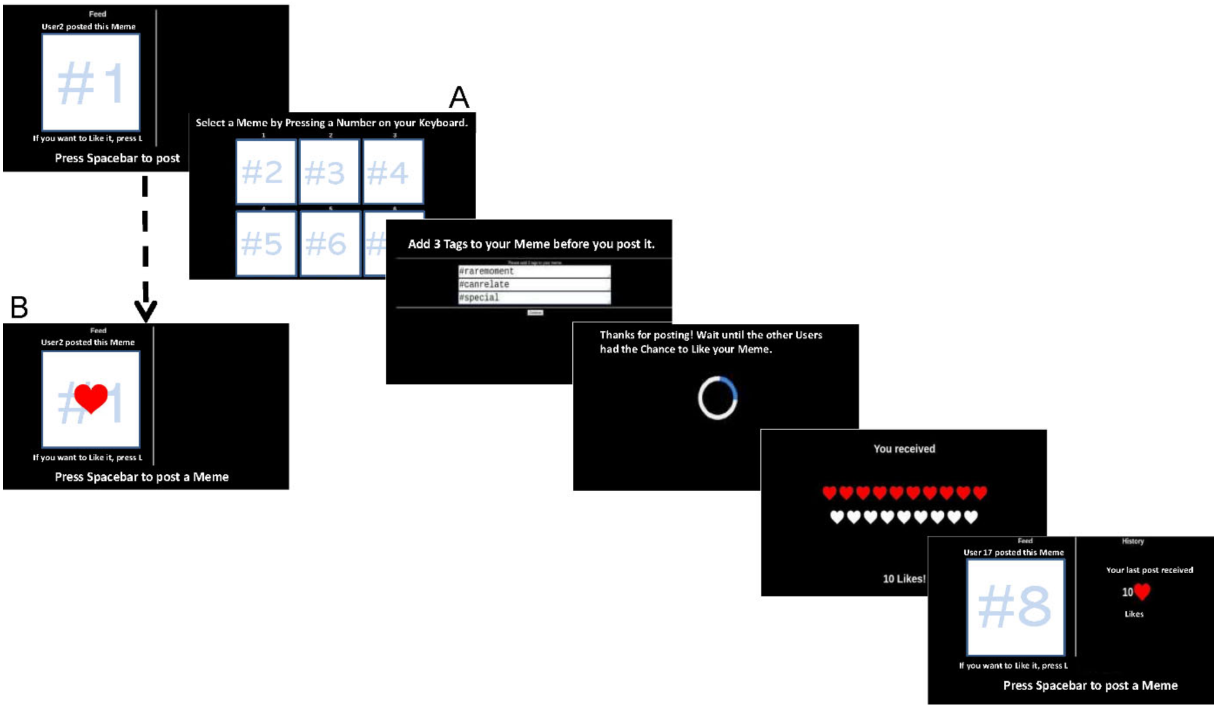

Supplementary Figure 5. Overview of the experimental design. Online participants could post "memes" (humorous pictures) (*A*) or provide likes for the memes posted by other ostensible users (*B*) whenever and as often they wished during 25 minutes. To post a meme, participants pressed the spacebar and then selected one from a random selection of 6 meme images. Participants were then asked to provide up to 3 "tags" (short keywords) to describe the selected meme. This was included to provide an analogue of the effort cost of posting on social media. Next, participants received feedback (likes, represented as filled hearts) on the posted meme. The average number of likes (4.5 vs 14.5 per post) was manipulated within participant (in counterbalanced order across participants) to test the influence of social rewards on posting response latencies. The number of likes received for the preceding post was displayed in the simulated "feed." Numbers ("#1") are placeholders for meme images due to copyright reasons.

Statistical analysis and data exclusions. We quantified response latencies with the interval from the first opportunity to post (indicated to the participant by the visual prompt "*Press Spacebar to post a Meme*") until the participant pressed the spacebar (see Supplementary Figure 4). To analyze the response latencies, we used multilevel GLMMs specifying a gamma distribution with a log link function (using the *glmmTMB* package[9]), as often recommended for response time data[10].

In the main analysis, data were excluded from three participants who spontaneously reported that they did not believe the likes were generated by real participants. Additionally, six data points with response latencies below 200 ms were excluded, as these are unlikely to reflect real posting decisions. This resulted in a final sample of 176 participants with 2,197 responses. Furthermore, we included the (mean-centered) proportion of completed tags per participant (mean proportion = 0.84, median proportion = 1) as an additive covariate, in order to control for effort variability. However, results remained the same regardless of these exclusions or modeling decisions (see Supplementary Note 12).

Supplementary Notes

Supplementary Note 1

To estimate the hyperbolic quantitative law of effect (QLE), we calculated the reward rate in twenty (Study 1, given the high average number of responses) or ten (Study 2, given the lower average number of responses) time bins for each individual, and assessed how the average response rate was predicted by the reward rate. We calculated the reward and response rates by time bin (rather than a fixed interval) to approximately equalize the number of data points between individuals. The QLE expresses the response rate (B) as a hyperbolic function of reward:

$$B = kR/(R + R_o) \qquad (1)$$

where k and R_O are a free parameters that determine the asymptote, and the reward to non-observed responses, respectively. We estimated the QLE for each individual using non-linear least squares, and compared the explained variance (expressed as the squared Pearson correlation between the predicted and actual values) to a linear model. The linear model consisted of a linear regression of response rates on reward rates. Both the QLE (k and R_O) and the linear model (intercept and slope) had two free parameters.

We found that the QLE explained the data better than the linear model in both Study 1 (mean R^2 QLE: 0.43, 99 % CI [0.41, 0.45], R^2 linear: 0.37, 99 % [0.36,0.39], paired t-test: t(2038) = 9.89, p < .0001), and Study 2 (pooled across datasets R^2 QLE: 0.37, 99 % CI [0.34, 0.38], R^2 linear: 0.34, 99 % [0.32, 0.36], t(2050) = 5.3, p < .0001). These results demonstrate

that aggregate behavior on social media is consistent with the well-established relationship between reward rates and response rates.

Supplementary Note 2

We report in the main text that Granger causality analyses of the empirical data showed that accrued likes "Granger cause" τ_{Post}. The lag number, which is the key analysis parameter specified for Granger causality analysis, was optimized based on analyses of simulated data. Specifically, we simulated generative models where the ground truth of causality ($\overline{\text{R}}$L model) and of *no* causality (No Learning model, and No Learning with Drift [NLD] model), respectively, was known, and applied Granger causality analysis to the simulated data in both cases.

These models represent different possible hypotheses about the mechanisms that generated the observed data. In the $\overline{\text{R}}$L model, τ_{Post} reflects a model policy (mean of an exponential distribution) that is dynamically updated to maximize accrued rewards (see "Methods" in the main text). In the No Learning model, τ_{Post} reflects a fixed response "policy" or tendency (mean of an exponential distribution) that is unrelated to, and thereby unaffected by, received reward. The No Learning model is used for model comparison in the main text. In the NLD model, the response tendency (mean on an exponential distribution) drifted (following a Gaussian random walk, with mean 0, and standard deviation σ. The stochastic nature of this formulation prevents accurate model estimation).

Before applying it to empirical data, we tuned the lag-number in the Granger causality analysis to correctly identify Granger causality in simulated data generated by the $\overline{\text{R}}$L model, and to reject Granger causality in data generated from the two models without learning. To this end, we simulated each of the models 1000 times (to approximate the size of the empirical datasets) with random parameter values from the unit range multiple times, and applied Granger causality analysis methods for panel data[11]. In the model simulations, we additionally varied whether the Poisson mean, which determined the amount of reward (i.e., likes), changed over time (see section "*Generative model simulations*" in the Supplementary Methods for details). Preliminary simulations demonstrated the importance of specifying a sufficiently long lag (i.e., the number of periods each time series is lagged for prediction) for the Granger causality analysis to avoid false positives. We found that setting the lag individually for each simulated individual to half of the maximal lag number given the constraints of the Granger causality function[11](i.e., $t_i/(5+3)/2$, where t is total number of posts for individual i), met this

criterion, while avoiding parameter aliasing. This specification consistently and correctly rejected Granger causality for both the No Learning and NLD models under varying parameter values, while consistently and correctly identifying Granger causality for simulations of the $\overline{R}$L model. Consequently, we used the same conservative lag specification for the Granger causality analyses of the empirical data reported in the main text. In summary, by applying the Granger causality method to simulated data where the ground-truth was known, we could fine-tune the statistical method for predictive causality estimation in a way that is not possible without generative models. This analysis showed that social rewards (i.e., likes) Granger caused τ_{Post} in all four empirical datasets (see main text).

Supplementary Note 3

We assessed the robustness of the model comparison results by evaluating two potential sources of bias: individuals with extreme average posting latencies (τ_{Post}), and especially few or many posts (i.e., data points for model fitting). As a conservative test, we removed all individuals that fell outside of the 20th – 80th percentiles on either variable, and assessed model fit in the remaining dataset.

We find that results, expressed as AIC_W for the $\overline{R}$L model, are highly similar to model comparison using the full datasets (c.f., main text): Study 1 (Instagram): Mean AIC_W = 0.69, 99 % CI [0.65, 0.71], t(1507) = 19. Study 2 (Men's Fashion): Mean AIC_W = 0.68, 99 % CI [0.64, 0.73], t(368) = 10.18, Study 2 (Women's Fashion): Mean AIC_W = 0.78, 99% CI [0.75, 0.82], t(517) = 20.63, p = 0, Study 2 (Gardening): Mean AIC_W = 0.83 99% [0.8, 0.87], t(554) = 26.6. In summary, these analyses show that the model comparison results, favoring the $\overline{R}$L model, are not dependent on outlier individuals, neither defined by τ_{Post} or total number of posts.

Supplementary Note 4

In the basic $\overline{R}$L – model, we assumed that the utility of likes followed an identity function (i.e., $u(R)$ = R). However, this might not be the case on social media such as Instagram, where posts can garner thousands of likes, which in turn might make users less sensitive or habituated to social rewards.

In order to investigate the utility function of likes on social media in more detail, we assessed three different, nested utility functions for R: (*i*) $u(R) = sR^d$, (*ii*) $u(R) = R^d$, (*iii*) $u(R) = sR$, where s ($0 \leq s \leq \infty$) and d ($0 \leq d \leq \infty$) are free parameters added to the $\overline{R}$L – model. As

evident, in formulations *i-ii*, the utility of *R* followed a nonlinear function that saturates more quickly for lower values of *d*, while *iii* simply scales *R* with *s*. Model comparison showed that model *ii*, which we refer to as the $\bar{R}L^{d}$- model, had the best fit (AICw: *i* = 0.15, *ii* = 0.51, *iii* = 0.34), and that the difference was unlikely to be due to chance (one sample t-test against P = 0.34: t(2,038) = 22.0, p < .0001, xp = 1). Notably, this formulation entails strongly diminishing marginal utility of *R* for values below 1 (1 = no discounting). For example, if an individual with *d* = 0.8 receives 100 likes, the effective utility is ~ 40, while for 1,000 likes, the utility is ~250. The mean *d* was reliably below 1 (M = 0.9, t(2,038) = 39.4, p < .0001), and with a skewed distribution (25th quantile = 0.006, 50th = 0.77, 75th = 1.35, 100th = 6.9), meaning that for most individuals, the marginal utility of likes was strongly diminishing. Adding the non-linear utility function $u(R) = R^{d}$ to the basic $\bar{R}$L – model better accounted for the data (AICw: 0.59 vs 0.41, xp = 1).

The relative improvement in model fit was positively associated with Instagram follower number (linear regression of log follower number on the difference in AICw relative to No Learning between $\bar{R}L^{d}$ and $\bar{R}$L – models: β = 0.013, SE = 0.003, t = 4.07, p < .0001. Regression of log follower number on AICw directly comparing $\bar{R}L^{d}$ and $\bar{R}$L – models: β = 0.011, SE = 0.004, t = 2.94, p = .003), indicating that incorporating diminishing marginal utility of likes improved model fit particularly for individuals with many followers. This suggests that individuals with many followers might have more strongly diminishing marginal utility functions. We tested this notion directly by predicting (log-transformed) follower number from the (standardized) estimated *d* parameter using linear regression (estimates were very similar when using negative-binomial regression, and when including all model parameters), controlling for differences in the number of posts (which otherwise might confound the relationship). We found that a lower estimated *d* parameter (i.e., more strongly diminishing marginal utility) was associated with a higher number of followers (β = -0.19, SE = 0.044, t = -4.16, p < .0001). In other words, the marginal utility of likes, which drives learning, was lower for individuals with more followers. Together, these results suggest that individuals with many followers may habituate to social rewards, necessitating more likes for an equivalent motivational effect. It should be noted that because the Instagram data was anonymized, we cannot rule out that individuals with many followers are qualitatively different in some unobserved way (e.g., represent businesses rather than private persons). However, the diminished marginal utility interpretation is in agreement with our experimental results, where

participants with more Instagram followers were less strongly affected by social rewards (see Supplementary Note 12).

We found no evidence for diminishing marginal utility in Study 2, as the basic $\overline{R}$L – model fit best in all three datasets (see Supplementary Table 2). This is likely due to the, on average, much lower number of likes per post in Study 2 than Study 1 (see Supplementary Table 1).

**Dataset**	***u(R) = R***	***u(R) = sR^d***	***u(R) = R^d***	***u(R)= sR***
Men's Fashion	0.42	0.15	0.26	0.15
Women's Fashion	0.46	0.08	0.27	0.19
Gardening	0.37	0.12	0.25	0.26

Supplementary Table 2. Comparison of utility functions in Study 2 showed, in contrast to Study 1, no evidence for diminishing marginal utility of likes in reinforcement learning. The table shows the relative evidence (AIC$_W$) for different utility functions added to the $\overline{R}$L – model. *u(R)* = R denotes the standard $\overline{R}$L – model. The exceedance probability (xp) for *u(R)* = R was 1 in all Study 2 datasets.

Supplementary Note 5

In our main analysis of Study 2, we only included posts with user-generated images, in order to be consistent with Study 1 (Instagram, where all posts are image based), and exclude directly communicative interactions (e.g., responding in writing to a comment directly aimed toward oneself). Image posts comprised 36% of the posts from the Men's Fashion dataset, 28% of posts from the Women's fashion dataset, and 20% of posts from the Gardening dataset.

To assess the effect of this decision, we repeated our primary model-based analysis but included all posts acquired for users with at least 10 image-based posts (i.e., the same users as in the main analysis). We find that the results hold when including all posts. The $\overline{R}$L model fit the data better than the No Learning model in all three datasets: Men's Fashion AIC$_W$ = 0.89 (one sample t-test against equal model fits: $t(542) = 36.78$, $p < .0001$), Women's Fashion AIC$_W$ = 0.88 ($t(772) = 40.62$, $p < .0001$), Gardening AIC$_W$ = 0.91 ($t(812) = 46.92$, $p < .0001$. All xp = 1).

Supplementary Note 6

To assess the specificity of the $\overline{\mathrm{R}}$L model, we conducted additional model comparisons that varied key features of the $\overline{\mathrm{R}}$L model. Finally, we compared the $\overline{\mathrm{R}}$L model to a model inspired by foraging theory.

Effect of time dependent terms. The $\overline{\mathrm{R}}$L model explicitly incorporates time-dependent effort and opportunity cost terms (see "*Description of $\bar{R}L$ model*" in main text) that scale with τ_{Post}. To ascertain that these terms, which were based on established theory for free-operant tasks[2], contributed to the explanatory power of the $\overline{\mathrm{R}}$L model, we compared it to two alternative models that did not include τ_{Post} - dependent terms. Both alternative models utilized the same policy gradient and likelihood function as the original model, but differed in how prediction errors and $\overline{\mathrm{R}}$ were computed. Here, we present model RL2:

$$\tau_{POST^t} = e^{Policy^t} \quad (2)$$

$$\Delta\tau_{Post^t} = \tau_{Post^t} - \tau_{Post^{t-1}} \quad (3)$$

$$\delta^t = R^t - C - \bar{R}^t \quad (4)$$

$$Policy^{t+1} = Policy^t + \alpha * \Delta\tau_{Post^t} * \delta^t \quad (5)$$

$$\bar{R}^{t+1} = \bar{R}^t + \alpha * \delta^t \quad (6)$$

In model RL3, the effort cost parameter C was removed, which simplifies equation Supplementary Equation 4 to:

$$\delta^t = R^t - \bar{R}^t \quad (7)$$

We compared the $\overline{\mathrm{R}}$L model to RL2 and RL3 using AICw. The $\overline{\mathrm{R}}$L model provided the best explanation of the data in all four datasets (all exceedance probabilities = 1, see Supplementary Table 3).

Dataset	$\overline{\mathrm{R}}$L model	RL2	RL3
Study 1: Instagram	0.69	0.09	0.22
Study 2: Men's Fashion	0.68	0.10	0.21
Study 2: Women's Fashion	0.71	0.15	0.14
Study 2: Gardening	0.76	0.08	0.16

Supplementary Table 3. Comparison of the $\overline{\mathrm{R}}$L model to alternative learning models without time-dependent effort- and opportunity cost terms. The table shows the mean AICw for each model. The exceedance probability (xp) for the $\overline{\mathrm{R}}$L model was 1 in all datasets.

Alternative effort cost formulations. The effort cost term of the $\overline{\text{R}}$L model (main text, eq. 2) is an exponentially decreasing function of τ_{Post}. This formulation is based on established RL theory for free operant tasks[2]. However, it is possible that the effort cost on social media takes different forms. We evaluated two alternative effort cost formulations: (i) exponentially increasing with time, and (ii) fixed. We did not find any evidence that either a fixed effort cost, or a cost that increased with post latency improved model fit (Supplementary Table 4).

For the Instagram dataset, the fit of the original $\overline{\text{R}}$L model and the model with increasing effort cost was similar (direct comparison: AIC_W = 0.502 vs 0.497, xp = .71). This might be because most users in the Instagram dataset posted with on average short latencies, which would render the influence of a time dependent (either positive or negative) effort cost term less pronounced. In line with this reasoning, we find that the original $\overline{\text{R}}$L model, with negative effort cost, fits relatively better for users with relatively longer average posting latencies, for whom the difference between the two effort cost variants would be most impactful on model fit (Spearman $\rho = 0.12$, $p < .0001$). In the three other datasets, where average posting latencies were longer (see Supplementary Table 1), we find that the original $\overline{\text{R}}$L model, where effort cost decreases with τ_{Post}, best explained the data (see Supplementary Table 4 below). We also find that the original decreasing effort cost formulation provides the overall best fit when the four datasets are pooled (combined AIC_W = 0.46, t-test against equal weights: ($t(4165) = 20.57$, $p < .0001$, xp = 1). Together, these results support the theory-based effort cost formulation of the $\overline{\text{R}}$L model, but suggest that the effort cost term is not critical for model fit, especially if the average τ_{Post} is short.

Dataset	$\overline{\text{R}}$L model	Fixed cost	Increasing cost
Study 1: Instagram	0.40/0.68	0.20/0	0.40/0.32
Study 2: Men's Fashion	0.41/1	0.32/0	0.27/0
Study 2: Women's Fashion	0.61/1	0.22/0	0.17/0
Study 2: Gardening	0.49/1	0.36/0	0.16/0

Supplementary Table 4. Comparison of the $\overline{\text{R}}$L model to alternative effort cost formulations. See Supplementary Note 7 for details. The table shows the mean AIC_W/exceedance probability for each model and dataset.

Effect of instrumental policy. A key component of the $\overline{\text{RL}}$ model is that it allows instrumental learning of the response policy (eq. 2-4, main text). In other words, the model can learn that slower/faster post latencies result in more reward, reflecting the hypothesis that social media users strategically adjust their rate of engagement to maximize social rewards. Alternatively, one can envision a "Pavlovian" policy, where the responses are faster following positive prediction errors ("approach") and slower following negative prediction errors ("avoidance"). We implemented this "Pavlovian Policy" model by changing the policy update equation (equation 4, main text) to:

$$Policy^{t+1} = Policy^{t} + \alpha * -\sqrt[3]{\delta^{t}} \quad (8)$$

In other words, the policy is directly updated with the (cube root of) the value prediction error. We take the cube root to reduce the influence of especially large prediction errors on the policy, which preliminary analysis showed was detrimental for model fit. Model comparison showed that the $\overline{\text{RL}}$ model explained the data best in all four datasets (t-tests against equal weights, largest p-value = .009, see Supplementary Table 5). This indicates that people instrumentally learn to maximize rewards by adjusting posting response latencies.

Dataset	$\overline{\text{RL}}$ model	Pavlovian Policy
Study 1: Instagram	0.62	0.38
Study 2: Men's Fashion	0.54	0.46
Study 2: Women's Fashion	0.61	0.39
Study 2: Gardening	0.56	0.44

Supplementary Table 5. Comparison of the $\overline{\text{RL}}$ model to alternative effort cost formulations. See text for details. The table shows the mean AICw for each model. The exceedance probability (xp) for the $\overline{\text{RL}}$ model was 1 in all datasets.

Comparison with a model based on foraging theory. Foraging theory provides a general framework for how organisms should maximize reward in decision making situations that extend over time[12]. Although foraging theory is more concerned with deriving optimal decision-making rules than with the precise mechanisms that produce behavior, some studies have successfully compared computational models based on foraging theory and RL[13]. Following this approach, we developed a stylized model inspired by the principles of foraging theory, in order to assess the specificity of the $\overline{\text{RL}}$ model as an explanation of reward

maximization on social media. A core principle of foraging theory is that organisms should maximize their net rate of intake by foraging in a given patch until the current reward rate falls below the average in the environment (marginal value theorem[14]). Because the social media environment involves many unobservables that would be required for a direct application of foraging theory and the marginal value theorem (e.g., travel time, different distinct patches, extended foraging bout[15]), our *F-model* is by necessity relatively abstract. We first describe the model, and then outline the relationship to foraging theory.

The core component of the F-model is the decision to forage (i.e., post) when the expected reward meets or exceeds a threshold T (i.e., $E(R)^t \geq T$). τ_{Post} follows an exponential distribution, given by:

$$\tau_{\text{Post}^t} \sim e^{E(R)^t - T} \tag{9}$$

where T is a free parameter ($0 \leq T \leq \infty$) that determines the threshold. $E(R)^t$ is a linear function of the time since the last post (t_{Last}) and the average reward rate, weighted by a free parameter P ($0 \leq P \leq \infty$). Practically, we solve for t by numerically searching for the root (i.e., 0) of the function $E(R)^t - T = 0$. Intuitively, $E(R)^t$ increases linearly with the time since the last post, with a slope given by the average reward rate:

$$E(R)^t = \bar{R}^t P t_{Last} \tag{10}$$

The average by unit time reward rate was calculated as a recency-weighted mean, with updating parameter α ($0 \leq \alpha \leq 1$) similar to the $\overline{\text{R}}$L model:

$$\bar{R}^{t+1} = \bar{R}^t + \alpha * \delta^t \tag{11}$$

$$\delta^t = R^t / \tau_{POST^t} - \bar{R}^t \tag{12}$$

The F-model is built on the assumption that the expected value of foraging (i.e., posting) goes to 0 directly after a post, and increases with time since the last post. In other words, likes are assumed to have a refractory time, and this refractory time is dependent on the average reward rate. The assumption that foraging reduces available reward is standard in foraging theory[16]. The F-model predicts, as the $\overline{\text{R}}$L model, that posting should be more frequent when the reward rate is high, as this maximizes the per unit time incurred reward.

The threshold parameter T can be interpreted in two ways that follow from foraging theory. First, it can be seen as an estimate of the overall average reward in the environment (which we cannot directly observe). Under this interpretation, the F-model decision rule (Supplementary Equation 9) is equivalent to the marginal value theorem: the forager should

leave the "patch" (a given social media platform, e.g., Instagram) if the expected value of foraging is lower than the average environmental reward, or conversely, forage in the patch if the expected value is higher than the average environmental value. A second interpretation is based on foraging theory for "sit and wait predators" that forage in one patch (rather than select between patches), and whose pray disperse after a foraging attempt (e.g., a school of small fish)[17–19]. For such predators, the optimal response time is equal to the refractory, or return, time of the prey[17–19]. Under this interpretation, T reflects the foragers estimated return time, or the time at which available reward (i.e., likes) returns to baseline.

We estimated the F-model, and found that while it provided a better explanation of the data than No Learning, the $\overline{\text{R}}$L model had a better fit in all four datasets (see Supplementary Table 6, all exceedance probabilities = 1). These results suggest that RL mechanisms provide a preferable account of the temporal dynamics of social media behavior.

**Dataset**	**$\overline{\text{R}}$L model**	**F-model**	**No Learning**
Study 1: Instagram	0.54	0.27	0.18
Study 2: Men's Fashion	0.47	0.34	0.19
Study 2: Women's Fashion	0.66	0.18	0.16
Study 2: Gardening	0.6	0.31	0.09

Supplementary Table 6. Comparison of the $\overline{\text{R}}$L model to the F-model. See Supplementary Note 7 for details. The table shows the mean AICw for each model. The exceedance probability (xp) for the $\overline{\text{R}}$L model was 1 in all datasets.

Supplementary Note 7

As in Study 1, the effect of $\overline{\text{R}}$ on τ_{Post} was larger for individuals for whom the RL model provided a better fit (interaction Low vs High $\overline{\text{R}}$ * AICw [centered at 0.5], Men's Fashion: $\beta = -0.12$, SE = 0.05, t = -2.9, p = .004, Women's Fashion: $\beta = -0.36$, SE = 0.08, t = -4.41, p < .0001, Gardening: $\beta = -0.29$, SE = 0.07, t = -4.34, p < .0001). This confirms the logic of the $\overline{\text{R}}$L model and our analysis approach.

Supplementary Note 8

To verify the robustness of the statistical results presented in the main text, we conducted additional analyses. The analyses of $\overline{\text{R}}$ reported in the main text are based on dichotomization

of the rank transformed and scaled (for each individual) $\overline{\mathrm{R}}$ variable. Here, we in addition report analyses with $\overline{\mathrm{R}}$ as a continuous term (either rank-transformed and standardized, or only standardized) (Supplementary Table 7). We also report the same set of results (including the analyses of the discrete High vs Low $\overline{\mathrm{R}}$ we report in the main text) using an alternative regression modeling approach based on cluster-robust standard errors (Supplementary Table 7). This methodology is more conservative than mixed-models and makes fewer assumptions. It is therefore popular in econometrics and adjacent fields[20]. We found that the different model formulations consistently showed that a higher estimated $\overline{\mathrm{R}}$ was predictive of shorter response latencies.

$\overline{R}$	**Model type**	**Dataset**	***B***	***SE***	***t***	***p***
Discrete - High vs Low	Cluster-corrected	Study 1: Instagram	-0.18	0.02	-8.11	< .0001
Continuous - Rank	Cluster-corrected	Study 1: Instagram	-0.11	0.014	-8.1	< .0001
Continuous - Rank	Mixed-model	Study 1: Instagram	-0.11	0.0017	-65.59	< .0001
Continuous - Z	Cluster-corrected	Study 1: Instagram	-0.09	0.0017	-53.9	< .0001
Continuous - Z	Mixed-model	Study 1: Instagram	-0.09	0.0017	-53.8	< .0001
Discrete - High vs Low	Cluster-corrected	Study 2: Men's Fashion	-0.1	0.03	-3.45	0.0005
Continuous - Rank	Cluster-corrected	Study 2: Men's Fashion	-0.08	0.017	-4.73	< .0001
Continuous - Rank	Mixed-model	Study 2: Men's Fashion	-0.06	0.009	-6.86	< .0001
Continuous - Z	Cluster-corrected	Study 2: Men's Fashion	-0.09	0.02	-4.93	< .0001
Continuous - Z	Mixed-model	Study 2: Men's Fashion	-0.053	0.009	-6.07	< .0001
Discrete - High vs Low	Cluster-corrected	Study 2: Women's Fashion	-0.21	0.04	-5.54	< .0001
Continuous - Rank	Cluster-corrected	Study 2: Women's Fashion	-0.16	0.02	-7.6	< .0001
Continuous - Rank	Mixed-model	Study 2: Women's Fashion	-0.12	0.016	-8.13	< .0001
Continuous - Z	Cluster-corrected	Study 2: Women's Fashion	-0.17	0.02	-7.58	< .0001

Continuous - Z	Mixed-model	Study 2: Women's Fashion	-0.11	0.017	-6.24	< .0001
Discrete - High vs Low	Cluster-corrected	Study 2: Gardening	-0.17	0.045	-3.7	0.0002
Continuous - Rank	Cluster-corrected	Study 2: Gardening	-0.11	0.03	-4.24	< .0001
Continuous - Rank	Mixed-model	Study 2: Gardening	-0.09	0.007	-12.64	< .0001
Continuous - Z	Cluster-corrected	Study 2: Gardening	-0.09	0.025	-3.73	0.0002
Continuous - Z	Mixed-model	Study 2: Gardening	-0.07	0.008	-9.62	< .0001

Supplementary Table 7. Regression estimates for the effect of $\overline{\mathrm{R}}$ in different regression models. The table shows the estimates for $\overline{\mathrm{R}}$, conditional on whether $\overline{\mathrm{R}}$ was dichotomized (as in the main text) or continuous (either rank transformed and then scaled within individual, or only scaled within individual), based on either log-linear mixed-models with a random effect for each user, or log-linear regression with cluster-robust standard errors. The effect was consistent in size and direction across the different model variations. P-values are not adjusted for multiple comparisons.

Supplementary Note 9

Previous research has shown that social comparison plays an important role in determining how many likes are required for a social media post to be experienced as successful[21], and that receiving fewer likes than close others generates negative affect[22]. Therefore, we asked whether social comparison[22] might account for additional variation in how reward learning mechanisms guide social media behavior. Because the format of the type of social media sites analyzed in Study 2 facilitates direct social comparison (one's post, and the likes it incurred, are displayed in sequential order together with others' posts on the same forum and topic), we focused our analysis on these datasets. As a model-based test for social comparison in reward learning, we modified the $\overline{\mathrm{R}}$L model to include an additional term, $-\xi \bar{R}_{Social}(t)$. Here, $\bar{R}_{Social}(t)$ refers to the median number of likes per post on the forum in the week preceding t, and ξ to a free parameter that determines the strength of social comparison. The social comparison term functions as a time-specific social reference level, which in practice can transform also large rewards into negative prediction errors if others on average receive even larger rewards. As these datasets lack information regarding the specific social information to which individual users attended, our test of social comparison is by necessity probabilistic. In other words, the model comparison tests how well the data adhered to patterns expected under a specific definition of social comparison.

This analysis suggested that social comparison may matter: the $\xi + \overline{\mathrm{R}}\mathrm{L}$ model explained posting dynamics better than the $\overline{\mathrm{R}}\mathrm{L}$ model (and the No Learning model) for the majority of users (mean AICw = 0.46, paired t-test of AICw for $\xi + \overline{\mathrm{R}}\mathrm{L}$ model vs AICw $\overline{\mathrm{R}}\mathrm{L}$ model: t(2,128) = 3.89, p = .0001), although this group-level evidence was relatively weak. Notably, social comparison in the $\xi + \overline{\mathrm{R}}\mathrm{L}$ model only occurs upwards (i.e., reflecting disadvantageous inequality or envy[23]): the rewards one receives become less valuable if others receive more[24]. Models that also included downward social comparison (advantageous inequality or pride/gloating[25]) provided an inferior account of the data—a pattern that further adheres to known dynamics of social comparison[20] (see Supplementary Table 8). Together, these exploratory results suggest that social comparison may contribute to reward learning dynamics on social media.

Alternative social comparison models. The $\xi + \overline{\mathrm{R}}\mathrm{L}$ model implements upwards social comparison (or disadvantageous inequality). We in addition tested two social comparison models that also included downward social comparison (advantageous inequality).

The first alternative social comparison model (ASC1) implemented a type of inequality aversion, parameterized with two free parameters (*a*,*β*), in addition to the three parameters of the basic $\overline{\mathrm{R}}\mathrm{L}$ model (equations 1-6, main text). In the ASC1, both downwards (*a*) and upwards (*β*) social comparison were defined in relation to the payoff of the individual:

$$\delta^t = R^t - \frac{C}{\tau_{Post^t}} - \bar{R}^t * \tau_{Post^t} + R^S \tag{13}$$

$$R^S = \begin{cases} \alpha(R^t - \bar{R}_{Social}{}^t), if R^t - \bar{R}_{Social}{}^t > 0 \\ \beta\left(R^t - \bar{R}_{Social}{}^t\right), if R^t - \bar{R}_{Social}{}^t < 0 \\ 0 \qquad\qquad\qquad\quad, if\ R^t - \bar{R}_{Social}{}^t = 0 \end{cases} \tag{14}$$

The second alternative social comparison model (ASC2) simplified ASC1, by allowing both forms of social comparison to be determined by one free parameter (ξ):

$$\delta^t = R^t - \frac{C}{\tau_{Post^t}} - \bar{R}^t * \tau_{Post^t} + \xi(R^t - \bar{R}_{Social}{}^t) \tag{15}$$

We compared the $\xi + \overline{\mathrm{R}}\mathrm{L}$ model to ASC1-ASC2 using AICw. The $\overline{\mathrm{R}}\mathrm{L}$ model provided the best explanation of the data in all three datasets of Study 2 (see Supplementary Table 8).

Dataset	$\xi + \overline{\mathrm{R}}\mathrm{L}$ model	ASC1	ASC2

Study 2: Men's Fashion	0.8	0.05	0.15
Study 2: Women's Fashion	0.74	0.06	0.2
Study 2: Gardening	0.6	0.19	0.21

Supplementary Table 8. Comparison of the $\xi + \overline{\text{R}}\text{L}$ model to alternative social comparison definitions. The table shows the mean AICw for each model. The $\xi + \overline{\text{R}}\text{L}$ model provided the best fit in all three datasets of Study 2 (all xp = 1).

Supplementary Note 10

Robustness analysis. Multiple quantitative criteria indicated that four clusters provided the best k-means cluster solution for the whole dataset (see main text). We assessed the robustness of this conclusion in two complimentary ways.

First, we randomly split the dataset into two equally sized partitions, and assessed the optimal number of clusters in each partition. We repeated this process ten times, and found that four clusters provided the best cluster solution in each of the 20 randomly determined partitions, which indicates that the four cluster solution reported in the main text was not determined by outliers or the exact sample composition.

Second, we randomly shuffled the rows of the dataset, independently for each column (i.e., for the 3 model parameters). This efficiently removed the pair-wise correlation between the model parameters (from $|r|$ = .15-.23 to .01), and should therefore eliminate the four "computational phenotypes" we identified (as these reflect different parameter value profiles). Indeed, we found that the shuffled dataset was best fit with a 3 cluster solution, where one cluster comprised 78% of all individuals (in contrast, the largest cluster in the original data only comprised 41% of the sample). This analysis indicates that the four cluster solution reported in the main text was not a structural necessity (as the shuffled data then should have the same structure and the same cluster solution), but unique to the four computational phenotype profiles. Together, these two analyses demonstrate both the stability and the specificity of the four social reward learning phenotypes.

Social comparison does not change cluster structure. We based the phenotyping analysis on the standard $\overline{\text{R}}\text{L}$ model, rather than the $\xi + \overline{\text{R}}\text{L}$ model that included social comparison, because we used the basic model to explain the data from both Study 1 (where no analysis of social comparison was possible) and Study 2 (where analysis of social comparison was possible). To rule out that omitting social comparison might have biased cluster assignment (e.g., that

individuals with a stronger tendency for social comparison should be clustered together), we performed two control analyses.

First, we conducted the same cluster analysis we report in the main text, but based on the $\xi + \overline{\text{RL}}$ model (for Study 1, we used the $\overline{\text{RL}}$ model parameters, and set $\xi = 0$) to assess whether inclusion of social comparison would change the cluster structure. This was not the case: multiple quantitative criteria supported a four cluster solution. Comparing cluster assignments to our original analysis (see main text), we find a strong agreement in cluster assignment (Cramer's V = 0.71, Rand index = 0.7). Furthermore, we find that the cluster centroids (the mean values of the parameters for each cluster) are extremely similar (correlation for the three overlapping model parameters: $r(10) = .99$, $p < .0001$). These results show that including social comparison does not change the cluster structure.

Second, we performed the same clustering analysis based on the $\xi + \overline{\text{RL}}$ model, but omitted the social comparison parameter ξ from the input features. This analysis controls for social comparison (as the other model parameters are adjusted for social comparison by the model estimation procedure), but does not use it for clustering. Again, we found that four clusters provided the best cluster solution. As for the preceding analysis, the cluster similarity relative to the clustering based on the standard $\overline{\text{RL}}$ model was high (Cramer V = 0.8, Rand index = 0.8), and centroids highly correlated ($r(10) = .99$, $p < .0001$). Together, these results show that basing the computational phenotyping on the $\overline{\text{RL}}$ model did not bias the results.

Supplementary Note 11

Robustness analyses. To assess the robustness of the experimental results from Study 3, we conducted a number of additional analyses. First, we assessed whether the direction with which the reward rate changed ("low to high" vs "high to low", counterbalanced across subjects) interacted with the reward rate condition, but did not find this to be reliably the case (Order*Reward rate: $\chi^2(1) = 2.18$, $p = 0.14$).

Second, we evaluated how our data inclusion and exclusion criteria affected the estimated experimental effect. Specifically, we tested whether removing the per participant percentage of completed "tags" covariate, which provides an index of effort cost, from the model affected the estimated effect. This was not the case; the effect of reward condition was almost identical without this covariate (reward condition: $\beta = 0.107$, SE = 0.044, $z = 2.43$, $p = 0.015$). Furthermore, we evaluated how the number of posts per participant influenced the

estimated effect of reward condition. In the main analysis, we included all participants with at least one post response. To test the robustness of this approach, we performed the same analyses for participants (N = 156) with at least 5 posts (reward condition: β = 0.11, SE = 0.045, z = 2.41, p = 0.016), or with at least one post in each reward condition (N = 167, reward condition: β = 0.105, SE = 0.044, z = 2.40, p = 0.016). Thus, in both cases, the effect of reward condition was comparable to the main analysis. Finally, we tested whether our decisions to remove (i) three participants who reported not believing that likes were generated by real participants, and (ii) six data points with response times shorter than 200 *ms* affected our results (see Supplementary Methods for description). We found that neither exclusion criteria had a substantial impact on the estimated experimental effect of reward condition (*i*: β = 0.103, SE = 0.044, z = 2.33, p = 0.019. *ii*: β = 0.108, SE = 0.045, z = 2.43, p = 0.015).

Participant-specific reward condition. Due to the random reward distribution and the relatively few responses per participant, the actual difference between high and low reward could differ markedly between individuals, which might lead to imprecise estimates. To account for this possibility, we repeated the analysis with by-participant median likes per reward condition as predictor. We found that using this semi-continuous predictor gave somewhat more precise results (β = -0.062, SE = 0.023, z = -2.72, p = 0.007) than the categorical reward condition predictor used in the main analyses, which corroborates the conclusion that changes in the social reward rate drives changes in response latency.

Analysis of individual differences. To assess if individual differences in self-reported real-life social media behavior moderated the experimental effect of social reward rate on posting response latencies, we analyzed the data from the subset of participants (n = 145) who opted to fill in all post experimental questionnaires. We first constructed a full regression model, and then simplified it using backwards elimination to identify the most important moderators. In the full model, we included; (*i*) the average number of likes received per social media post, (*ii*) number of followers on Instagram, (*iii*) number of followers on Twitter, (*iv*) number of followers on Facebook, and (*v*) the 9 item version of the *Social Media Disorder Scale*[8], all in interaction with reward condition. All continuous predictors were mean-centered. After deletion, the final model included only reward condition (main effect: β = 0.118, SE = 0.049, z = 2.39, p = 0.017) in interaction with *ii*, the number of followers on Instagram. Intriguingly, a larger number of followers on Instagram was associated with a weaker effect of reward condition (Instagram followers * Reward condition interaction: β = -0.0016, SE = 0.0006, z = -2.62, p = 0.009). This parallels our finding that having more Instagram followers was

associated with more strongly diminishing marginal utility of likes in Study 1 (see Supplementary Note 5) Repeating the analysis with the semi-continuous participant specific median likes predictor (see "*Participant-specific reward condition*" in Supplementary Note 12) gave comparable results (Instagram followers * median reward interaction β = 0.0011, SE = 0.0003, z = 3.54, p = 0.0004).

$\overline{\text{R}}$L model-based analysis. For consistency with our main results, we estimated the $\overline{\text{R}}$L model for participants with at least 5/10 (n = 156/97) responses to generate $\overline{\text{R}}$ time series (which were converted to High vs Low $\overline{\text{R}}$ identically to the main analysis). Naturally, estimating a model with a limited number of data points (e.g., 5-33 in the experiment instead of 10-11649 in Study 1) is prone to high variance and overfitting. Nonetheless, by utilizing the model for latent variable inference[26] with multi-level regression (which reduces variance by shrinkage) instead of estimation-based inference, this analysis provides converging evidence that the social reward rate drives behavior, consistent with our theoretical account.

In the main text, we report the direct effect of High vs Low $\overline{\text{R}}$ on response latencies (i.e., without the categorical reward condition predictor) for participants with at least 5 responses. By adding both the model-derived and the experiment-based predictor to the model, we next evaluated the shared explanatory value. We found that both the model-derived and experiment-based estimates are reduced in magnitude (0.284 to 0.274, and 0.109 to 0.076, respectively), and that only the model-derived regressor remains conventionally significant (model-derived: z = 6.0, p < .0001. Experimental: z = 1.69, p = .09). This indicates that the regressors, as expected, partially explained the same variance, but that the individual specific model-derived regressor better predicts response latencies. Finally, we repeated the analysis for participants with at least 10 responses (as in our analysis of Study 1-2), and find results to be comparable (n = 97, β = 0.31, SE = 0.052, z = 6.18, p < .0001).

Supplementary Note 12

Because the Instagram dataset used in Study 1 was based on participation in a photography contest on Instagram (see "*Additional information about Study 1*" in the Supplementary Methods for details), we tested whether the number of such contest participations, as indexed by the number of posts with "*#whp-*" hashtags, was associated with social media behavior (as measured by our $\overline{\text{R}}$L model). We predicted (log) "*#whp-*" tag number from the estimated parameters of the $\overline{\text{R}}$L model, together with the total number of posts (which naturally is the most important predictor) using linear regression. We found no evidence that the number of

“*#whp-*“ tags was associated with $\overline{\text{RL}}$ parameters (neither using the basic $\overline{\text{RL}}$ model or the $\overline{\text{RL}}$ model augmented with a non-linear utility function): the lowest *p*-value for any estimated parameter was ~0.25. Together, these results indicate that contest participation did not have any clear influence on posting behavior, and thus our results are likely to generalize beyond this context (as further suggested by Study 2 results).

Supplementary References

1. Ferrara, E., Interdonato, R. & Tagarelli, A. Online popularity and topical interests through the lens of instagram. in *Proceedings of the 25th ACM conference on Hypertext and social media - HT ’14* 24–34 (ACM Press, 2014). doi:10.1145/2631775.2631808

2. Niv, Y., Daw, N. D., Joel, D. & Dayan, P. Tonic dopamine: opportunity costs and the control of response vigor. *Psychopharmacology (Berl).* **191,** 507–520 (2007).

3. Staddon, J. E. R. *Adaptive Behavior and Learning: Second Edition*. *Adaptive Behavior and Learning: Second Edition* (Cambridge University Press, 2016). doi:10.1017/CBO9781139998369

4. Freestone, D. M., Balci, F., Simen, P. & Church, R. M. Optimal response rates in humans and rats. *J. Exp. Psychol. Anim. Learn. Cogn.* **41,** 39–51 (2015).

5. Gibbon, J. Scalar expectancy theory and Weber’s law in animal timing. *Psychol. Rev.* **84,** 279–325 (1977).

6. Wagenmakers, E.-J. & Farrell, S. AIC model selection using Akaike weights. *Psychon. Bull. Rev.* **11,** 192–6 (2004).

7. Daunizeau, J. *et al.* VBA: A Probabilistic Treatment of Nonlinear Models for Neurobiological and Behavioural Data. *PLoS Comput. Biol.* **10,** e1003441 (2014).

8. Van Den Eijnden, R. J. J. M., Lemmens, J. S. & Valkenburg, P. M. The Social Media Disorder Scale: Validity and psychometric properties. *Comput. Human Behav.* **61,** 478–487 (2016).

9. glmmTMB citation info. Available at: https://cran.r-project.org/web/packages/glmmTMB/citation.html. (Accessed: 21st February 2020)

10. Ng, V. K. Y. & Cribbie, R. A. Using the Gamma Generalized Linear Model for Modeling Continuous, Skewed and Heteroscedastic Outcomes in Psychology. *Curr. Psychol.* **36,** 225–235 (2017).

11. Croissant, Y. & Millo, G. Panel Data Econometrics in *R* : The **plm** Package. *J. Stat. Softw.* **27,** 1–43 (2008).

12. Pyke, G. H., Pulliam, H. R. & Charnov, E. L. Optimal Foraging: A Selective Review

of Theory and Tests. *Q. Rev. Biol.* **52,** 137–154 (1977).

13. Constantino, S. M. & Daw, N. D. Learning the opportunity cost of time in a patch-foraging task. *Cogn. Affect. Behav. Neurosci.* **15,** 837–853 (2015).

14. Charnov, E. L. Optimal foraging, the marginal value theorem. *Theor. Popul. Biol.* **9,** 129–136 (1976).

15. Gabay, A. S. & Apps, M. A. J. Foraging Optimally in Social Neuroscience: Computations and Methodological considerations. doi:10.31234/OSF.IO/FUD9P

16. Pyke, G. H. *OPTIMAL FORAGING THEORY: A CRITICAL REVIEW. Ann. Rev. Ecol. Syst* **15,** (1984).

17. Katz, M. W. *et al. A predator-prey foraging game: how does prey density influence tactics?* (2014).

18. Hugie, D. M. The waiting game: a 'battle of waits' between predator and prey. *Behav. Ecol.* **14,** 807–817 (2003).

19. Wacht Katz, M., Abramsky, Z., Kotler, B., Altstein, O. & Rosenzweig, M. L. *Playing the waiting game: predator and prey in a test environment*. (2010).

20. Colin Cameron, A. & Miller, D. L. A Practitioner's Guide to Cluster-Robust Inference. *J. Hum. Resour.* **50,** 317–372 (2015).

21. Carr, C. T., Hayes, R. A. & Sumner, E. M. Predicting a Threshold of Perceived Facebook Post Success via Likes and Reactions: A Test of Explanatory Mechanisms. *Commun. Res. Reports* **35,** 141–151 (2018).

22. Rosenthal-von der Pütten, A. M. *et al.* "Likes" as social rewards: Their role in online social comparison and decisions to like other People's selfies. *Comput. Human Behav.* **92,** 76–86 (2019).

23. Fehr, E. & Schmidt, K. M. A Theory of Fairness, Competition, and Cooperation. *Q. J. Econ.* **114,** 817–868 (1999).

24. Fliessbach, K. *et al.* Social Comparison Affects Reward-Related Brain Activity in the Human Ventral Striatum. *Science (80-.).* **318,** 1305–1308 (2007).

25. Wills, T. A. Downward comparison principles in social psychology. *Psychol. Bull.* **90,** 245–271 (1981).

26. Wilson, R. C. & Collins, A. G. E. Ten simple rules for the computational modeling of behavioral data. *Elife* **8,** (2019).
