# Supplementary material for: A computational reward learning account of social media engagement
Source: Nat Commun. 2021 Feb 26;12:1311. doi: 10.1038/s41467-020-19607-x (PMC7910435; doi:10.1038/s41467-020-19607-x)
Supplement: Supplementary file 3 — Reporting Summary [file 41467_2020_19607_MOESM3_ESM.pdf]

nature research

Corresponding author(s): Björn Lindström

Last updated by author(s): 23/02/21

Reporting Summary

Nature Research wishes to improve the reproducibility of the work that we publish. This form provides structure for consistency and transparency in reporting. For further information on Nature Research policies, see Authors & Referees and the Editorial Policy Checklist.

Statistics

For all statistical analyses, confirm that the following items are present in the figure legend, table legend, main text, or Methods section.

n/a	Confirmed	
☐	☒	The exact sample size (n) for each experimental group/condition, given as a discrete number and unit of measurement
☐	☒	A statement on whether measurements were taken from distinct samples or whether the same sample was measured repeatedly
☐	☒	The statistical test(s) used AND whether they are one- or two-sided<br>*Only common tests should be described solely by name; describe more complex techniques in the Methods section.*
☐	☒	A description of all covariates tested
☐	☒	A description of any assumptions or corrections, such as tests of normality and adjustment for multiple comparisons
☐	☒	A full description of the statistical parameters including central tendency (e.g. means) or other basic estimates (e.g. regression coefficient) AND variation (e.g. standard deviation) or associated estimates of uncertainty (e.g. confidence intervals)
☐	☒	For null hypothesis testing, the test statistic (e.g. F, t, r) with confidence intervals, effect sizes, degrees of freedom and P value noted<br>*Give P values as exact values whenever suitable.*
☒	☐	For Bayesian analysis, information on the choice of priors and Markov chain Monte Carlo settings
☒	☐	For hierarchical and complex designs, identification of the appropriate level for tests and full reporting of outcomes
☒	☐	Estimates of effect sizes (e.g. Cohen's d, Pearson's r), indicating how they were calculated

Our web collection on statistics for biologists contains articles on many of the points above.

Software and code

Policy information about availability of computer code

Data collection	R 3.5.1 was used to obtain web-based data in Study 2. PsiTurk (2.3.9) was used for experimental data collection for Study 3. Data are available on the Open Science Framework (https://osf.io/765py/).
Data analysis	R 3.5.1 was used for all data analyses. The lme4 (1.1-23), lmerTest (3.1-2) and glmmTMB (1.0.1) packages were used for mixed effects modeling. The plm package (2.2-3) was used for panel Granger analysis. The NbClust (3.0) package was used for determining optimal number of clusters for k-means clustering.<br><br>Model code is available on the Open Science Framework (https://osf.io/765py/).

For manuscripts utilizing custom algorithms or software that are central to the research but not yet described in published literature, software must be made available to editors/reviewers. We strongly encourage code deposition in a community repository (e.g. GitHub). See the Nature Research guidelines for submitting code & software for further information.

Data

Policy information about availability of data

All manuscripts must include a data availability statement. This statement should provide the following information, where applicable:

- Accession codes, unique identifiers, or web links for publicly available datasets
- A list of figures that have associated raw data
- A description of any restrictions on data availability

The availability of the dataset analyzed in Study 1 is described in Ferrara, E., Interdonato, R. & Tagarelli, A. Online popularity and topical interests through the lens of instagram. in Proceedings of the 25th ACM conference on Hypertext and social media - HT '14 24–34 (ACM Press, 2014). doi:10.1145/2631775.2631808.

The datasets analyzed in Study 2 are available on reasonable request from the corresponding author. The dataset analyzed in Study 3 is available on the Open Science Framework (https://osf.io/765py/).

Field-specific reporting

Please select the one below that is the best fit for your research. If you are not sure, read the appropriate sections before making your selection.

☐ Life sciences ☒ Behavioural & social sciences ☐ Ecological, evolutionary & environmental sciences

For a reference copy of the document with all sections, see nature.com/documents/nr-reporting-summary-flat.pdf

Behavioural & social sciences study design

All studies must disclose on these points even when the disclosure is negative.

Study description

The research is based on quantitative analysis of social media data (Study 1-2), and experimental data collected online (Study 3).

Research sample

Study 1 was based on previously published publicly available data (described in Ferrara E, Interdonato R, Tagarelli A (2014) Online popularity and topical interests through the lens of instagram. Proceedings of the 25th ACM Conference on Hypertext and Social Media - HT '14 (ACM Press, New York, New York, USA), pp 24–34.).

For Study 2, we obtained publicly accessible data from three different topic-focused social media discussion forums (men's fashion: styleforum.net, women's fashion: forum.purseblog.com, gardening: garden.org). The final datasets were fully anonymized, and only included the time stamps and likes of each post.

For Study 3, we invited 200 participants (with minimum 95% approval rate) on Amazon Mechanical Turk to take part in a study on "humor on social media". 179 participants completed the study, and were payed 3$ in compensation. Because the purpose of the Study 3 was as proof of principle, we recruited a non-representative convenience sample from the general population. The study was approved by the ethical review board of the University of Amsterdam, The Netherlands. All participants provided informed consent.

Sampling strategy

Study 1 was based on a publicly available dataset, containing a random sample of individuals who partook in a specific photography contest on Instagram in 2014 (for further information, see Ferrara E, Interdonato R, Tagarelli A (2014) Online popularity and topical interests through the lens of instagram. Proceedings of the 25th ACM Conference on Hypertext and Social Media - HT '14 (ACM Press, New York, New York, USA), pp 24–34).

For Study 2, we obtained publicly available data (i.e., post time stamps and likes) from three topic focused social media forums (men's fashion: styleforum.net, women's fashion: forum.purseblog.com, gardening: garden.org), where users could provide each other likes as feedback to others posts. These forums are organized in "threads" (which users can start) focused on a specific topic or question. Because our question concerned how likes affect posting behavior, we for simplicity focused on threads with a high proportion of image posts rather than textual exchange (where many other factors than likes are likely to affect behavior). For this purpose, we selected three high profile threads (with many thousands of posts each), where users primarily posted images of their own clothes, from the men's fashion forum, and eight threads (primarily on topics related to posting images of users' handbags or shoes) from the women's fashion forum. On the gardening forum, we acquired all posts from the entire forum, because the forum allowed likes from its start (which allowed tracking the entire learning history of individual users. In the two other forums, we only analyzed posts from the time period where likes were possible). The similarity of results from Instagram (Study 1) and the two fashion forums (where the analyses was based on a subset of threads, Study 2), and from the gardening forum (where the analyses included all threads, Study 2), indicates that the results are robust to variation in data sampling strategy.

For the Study 3, we invited 200 participants (with minimum 95% approval rate) on Amazon Mechanical Turk to take part in a study on "humor on social media." 179 participants completed the study. Approximate sample size calculation was based on the data from Study 1, which showed that the effect of High vs Low social reward rate on response latencies was significant at the 5% level in 20 out of 20 random (with replacement) subsamples of 200 individuals (also when limiting the number of data points for each individual to a number plausible for an experiment).

Data collection

Data collection for Study 1 was conducted by Ferrara and colleagues (described in Ferrara E, Interdonato R, Tagarelli A (2014) Online popularity and topical interests through the lens of instagram. Proceedings of the 25th ACM Conference on Hypertext and Social Media - HT '14 (ACM Press, New York, New York, USA), pp 24–34.). Data collection for Study 2 was conducted with custom written R 3.5.1 functions applied to publicly accessible data. The final datasets were fully anonymized, and only retained time stamps and likes (see "Sampling strategy").

Data collection for Study 3 was conducted on Amazon Mechanical Turk, using PsiTurk. The participants conducted the computerized experiment in their home setting on their own computers, with no experimenter present. Data was collected automatically by the web server. The experimenter was not blinded to the experimental condition, because the experimenter never interacted with the participants.

Timing

The dataset used for Study 1 was collected in 2014 (for further information, see Ferrara E, Interdonato R, Tagarelli A (2014) Online popularity and topical interests through the lens of instagram. Proceedings of the 25th ACM Conference on Hypertext and Social Media - HT '14 (ACM Press, New York, New York, USA), pp 24–34.)

The data used in Study 2 were acquired in January 2019 (Study 2 A), January 2019 (Study 2 B), November 2018 (Study 2 C).

The data used in Study 3 was collected in January 2020.

Data exclusions

In all datasets of Studies 1-2, we excluded users with fewer than 10 posts, in order to allow model-based analysis of learning effects

Data exclusions	(determined based on Schulz, E., Bhui, R., Love, B.C., Brier, B,., Todd, M.T., & Gershman, S.J. (2019). Structured, uncertainty-driven exploration in real-world consumer choice. Proceedings of the National Academy of Sciences, 116, 13903-13908). In Study 1, this excluded 4 individuals. In Study 2A, this excluded 1399 users, in Study 2B 3850 users, and in Study 2C 697 users.<br><br>For the main analysis in Study 3, data were excluded from three participants (out of 179) who spontaneously reported that they did not believe the likes were generated by real participants. Additionally, six individual data points with response latencies below 200 ms were excluded, as these are unlikely to reflect real posting decisions. This resulted in a final sample of 176 participants with 2197 responses. Furthermore, we included the (mean-centered) proportion of completed tags per participant (mean proportion = 0.84, median proportion = 1) as an additive covariate, in order to control for effort variability. We did not preregister these exclusions, but verify that they did not impact our conclusions (see “Additional analysis of experimental results” in the Supplementary Material).
Non-participation	No participants dropped out of Study 3.
Randomization	Study 3 used a within individual design, with the order of conditions randomized.

Reporting for specific materials, systems and methods

We require information from authors about some types of materials, experimental systems and methods used in many studies. Here, indicate whether each material, system or method listed is relevant to your study. If you are not sure if a list item applies to your research, read the appropriate section before selecting a response.

Materials & experimental systems

n/a	Involved in the study	
☒	☐	Antibodies
☒	☐	Eukaryotic cell lines
☒	☐	Palaeontology
☒	☐	Animals and other organisms
☐	☒	Human research participants
☒	☐	Clinical data

Methods

n/a	Involved in the study	
☒	☐	ChIP-seq
☒	☐	Flow cytometry
☒	☐	MRI-based neuroimaging

Human research participants

Policy information about studies involving human research participants

Population characteristics	See above for sample information. All participants in the experiment were above 18 years of age. Demographic information was not mandatory to report for the participants, hence full sample demographics are not available.
Recruitment	For Study 3, we invited 200 participants (with minimum 95% approval rate) on Amazon Mechanical Turk to take part in a study on “humor on social media”. We are not aware of any possible sources of self-selection.
Ethics oversight	The experimental study (Study 3) was approved by the ethical review board of the University of Amsterdam, The Netherlands.

Note that full information on the approval of the study protocol must also be provided in the manuscript.
